# No excess risk of adverse pregnancy outcomes among women with serological markers of previous infection with *Coxiella burnetii*: evidence from the Danish National Birth Cohort

**DOI:** 10.1186/1471-2334-13-87

**Published:** 2013-02-17

**Authors:** Stine Yde Nielsen, Anne-Marie Nybo Andersen, Kåre Mølbak, Niels Henrik Hjøllund, Bjørn Kantsø, Karen Angeliki Krogfelt, Tine Brink Henriksen

**Affiliations:** 1 Department of Occupational Medicine, Regional Hospital West Jutland, Gl. Landevej 61, Herning 7400, Denmark; 2 Perinatal Epidemiology Research Unit, Aarhus University Hospital, Skejby, Brendstrupgaardsvej, Aarhus N, Denmark; 3 Section of Social Medicine, Department of Public Health, University of Copenhagen, Oster Farimagsgade 5, Copenhagen, DK-1014, Denmark; 4 Department of Infectious Disease Epidemiology, Statens Serum Institut, Artillerivej 5, Copenhagen S, 2300, Denmark; 5 Department of Clinical Epidemiology, Aarhus University Hospital, Aarhus, Denmark; 6 Department of microbiology and infection control, Statens Serum Institut, Artillerivej 5, Copenhagen S, 2300, Denmark; 7 Department of microbiology and infection control, Statens Serum Institut, Artillerivej 5, Copenhagen S, 2300, Denmmark; 8 Perinatal Epidemiology Research Unit and Department of Pediatrics, Aarhus University Hospital, Brendstrupgaardsvej, Aarhus N, 8200, Denmark

**Keywords:** Q fever, *Coxiella burnetii*, Infection, Human, Pregnancy, Spontaneous abortion, Preterm birth

## Abstract

**Background:**

Q fever caused by *Coxiella burnetii* is transmitted to humans by inhalation of aerosols from animal birth products. Q fever in pregnancy is suspected to be a potential cause of fetal and maternal morbidity and fetal mortality but the pathogenesis is poorly understood, and even in Q fever endemic areas, the magnitude of a potential association is not established.

We aimed to examine if presence of antibodies to *C. burnetii* during pregnancy or seroconversion were associated with adverse pregnancy outcomes.

**Methods:**

The Danish National Birth Cohort collected blood samples and interview data from 100,418 pregnant women (1996–2002). We sampled 397 pregnant women with occupational or domestic exposure to cattle or sheep and a random sample of 459 women with no animal exposure. Outcome measures were spontaneous abortion, preterm birth, birth weight and Small for Gestational Age (SGA).

Blood samples collected in pregnancy were screened for antibodies against *C. burnetii* by enzyme-linked immunosorbent assay (ELISA). Samples positive for IgG or IgM antibodies in the ELISA were confirmed by immunofluorescence antibody test (IFA).

**Results:**

Among the 856 women, 169 (19.7%) women were IFA positive; 147 (87%) of these had occupational or domestic contact with livestock (IFA cutoff > =1:128).

Two abortions were IFA positive vs. 6 IFA negative (OR: 1.5; 95%CI: 0.3-7.6). Three preterm births were IFA positive vs. 38 IFA negative (OR: 0.4; 95% CI: 0.1-1.1). There was a significant difference in birth weight of 168 g (95% CI: 70-267 g) with IFA positive being heavier, and the risk of being SGA was not increased in the newborns of IFA positive women (OR: 0.4; 95%CI: 0.8-1.0).

Most seropositive women were IgG positive indicating previous exposure. Seroconversion during pregnancy was found in 10 women; they all delivered live babies at term, but two were SGA.

**Conclusion:**

We found no increased risk of adverse pregnancy outcome in women with verified exposure to *C. burnetii*.

To our knowledge, this is the first population-based seroepidemiologic study evaluating pregnancy outcome in women with serologically verified exposure to *C. burnetii* against a comparable reference group of seronegative women.

## Background

Q fever is a zoonotic infection caused by *Coxiella burnetii,* an intracellular pathogen. In small ruminants Q fever is known to cause abortions, retained placenta, endometritis and infertility. Placentas of infected animals contain high numbers of bacteria [1,2]; the bacteria remain viable for months in the environment.

Human infection is usually acquired through inhalation of contaminated aerosols from infected animals that contaminate the environment in particular through excretion of the bacteria in large amounts in birth-by-products, especially placenta [3-5].

Q fever has previously been considered a rare, imported infection in Denmark, but recent studies have found antibodies against *C. burnetii* in a large percentage of Danish dairy cattle as well as in humans exposed to livestock [6-8].

For otherwise healthy people, Q fever infection is often asymptomatic or has a mild, flu-like course, but may also cause severe pneumonia. Pregnant women, immunocompromised patients and patients with pre-existing cardiac valve- or vascular defects are at risk of a severe course of infection [3,5].

Q fever in pregnancy is suspected to be a potential cause of fetal morbidity and mortality, but the pathogenesis is poorly understood, and even in Q fever endemic areas the magnitude of a potential association is not established.

Present evidence mainly originates from French case studies of referred infected pregnant patients in which untreated infection was followed by spontaneous abortion, intrauterine growth retardation, oligohydramnion, stillbirth or premature delivery [9]. Infection in pregnancy is often asymptomatic but may imply an increased risk of chronic infection and a risk of reactivation of a past infection in subsequent pregnancies has been suggested [9-11].

Two new studies evaluated infection in pregnancy and found no increased risk of adverse pregnancy outcome in seropositive pregnancies [12,13].

Although Q fever is endemic worldwide, the reported prevalence seems to be highest in areas with medical or scientific awareness of the infection and many obstetricians know little about the infection [10]. Since the evidence of pregnancy outcome in women with Q fever infection relies primarily on case reports, unbiased estimates of the risks of adverse pregnancy outcome among infected women remain largely unknown.

Our primary objectives were to evaluate the association between antibodies to *C. burnetii* and pregnancy outcome and to compare pregnancy outcome in women who seroconverted during pregnancy with seronegative pregnant women.

## Methods

### Participants

The study was based on interview data and blood samples from the Danish National Birth Cohort (DNBC), which is a nationwide cohort of 100,418 pregnant women and their offspring.

Enrolment in the DNBC took place between 1996 and 2002. The women were recruited in connection with the first antenatal visit to the general practitioner. Information on variables reflecting exposures before and during the early part of pregnancy was collected by means of a computer assisted telephone interview scheduled around gestational week 12. A second interview was scheduled in week 30 (interview forms are available at the website for the cohort).

During pregnancy, two blood samples were collected; one between gestational weeks 6 to 12, the second in gestational week 24. A sample was also drawn from the umbilical cord.

The interviews were performed if the women were reached within four phone calls, and if they agreed to participate.

The interviews covered reproductive history, age, smoking status, domestic contact to animals as well as very detailed questions regarding occupational contact to different animals.

A detailed description of the cohort can be found elsewhere [14].

In women who participated in the first interview and who also provided a blood sample (n = 95000) the study population was defined as follows: Occupational contact with livestock (n = 195), domestic contact with cattle or sheep (n = 202) and a randomly selected sample with no contact to livestock (n = 459). Blood samples from these 856 women were analyzed for antibodies against *C. burnetii.*

Pregnancy outcome was defined as:

Spontaneous abortion: fetal loss before 154 days (22 weeks) after the first day of the last menstrual period with gestational age estimated from the participants’ self- reported last menstrual period.

Preterm delivery: delivery (live births and stillbirths) between gestational weeks 22 + 0 days and 36 weeks + 6 days.

Small for gestational Age (SGA): for children born from week 37 + 0 and onwards, SGA was defined as a birth weight corresponding to the 10^th^ percentile in gram and below. Children with a birth weight above the 10^th^ percentile were used as reference group.

The relationship between serological status, birth weight and gestational age, respectively, was also evaluated.

We also evaluated late induced abortions and stillbirth.

### Detection of antibodies against C. burnetii

*C. burnetii* expresses two antigens, phase I and phase II. When infected, phase II IgG and IgM antibodies are elevated, and they may remain positive for months to years. A large study from Australia and England found that phase II IgG antibodies persisted after four and 12 years, respectively [15].

In acute Q fever, primarily antibodies against phase II are raised, and titers are higher than antibodies against phase I. As with most other infections, IgM antibodies appear first.

In chronic forms of the disease, antibodies against phase I are elevated.

In order to determine antibodies against *C. burnetii,* we chose a two-step approach. First, all samples were screened in a commercial enzyme-linked immunosorbent assay (ELISA). Positive samples from the ELISA were confirmed with an immunofluorescence antibody test (IFA) which is considered to be gold standard when diagnosing Q fever.

The commercial ELISA kit were purchased from Panbio (Queensland, Australia) (cat. no. E-QFB01G and E-QFB01M) and used according to the manufacturer’s instructions with minor modifications; due to low sample volume the samples were diluted differently from what was prescribed in the instructions but the same dilution factors were used.

Samples which were positive for either IgG or IgM antibodies in the ELISA were confirmed with an IFA test from Focus Diagnostics (ca. no. IF0200G and IF0200M). The test was performed according to the instructions provided by the manufacturer, with the following minor modifications: due to a low amount of sample material, the diluted samples 1:10 from the ELISA were used to further dilute the samples as described by the manufacturer. The effect of the dilution in the Panbio buffer was tested prior to the use on patient samples and did not show any influence on the results (results not shown).

A local cutoff adjusted to the Danish population has been defined [16], including negative, equivocal and positive titers. When the ELISA positive samples in our study were reanalyzed using IFA, a modified version of the Danish cutoff was used. A sample was considered IFA positive when any of the phases were 1:128 or above.

For women without animal exposure, only the blood sample from the first trimester was analyzed. In women with contact to livestock, blood samples from the umbilical cord or mid-pregnancy were analyzed initially (n = 361 women) and therefore seroconversion during pregnancy could be monitored.

In order to detect a possible seroconversion throughout pregnancy, our strategy was to initially analyze the last existing blood sample (for 79 women this was the mid pregnancy sample and for 282 it was the umbilical cord sample). If this sample was tested positive in ELISA, the first blood sample from pregnancy week 12–16 was analyzed using ELISA.

In order to select which of the ELISA positive samples from the beginning of pregnancy were to be reanalyzed in IFA, the following criteria had to be met: a change in ELISA from negative in the beginning of pregnancy to positive in the mid-pregnancy or umbilical cord sample or a doubling in the adjusted ELISA OD-value throughout pregnancy.

In analyses of pregnancy outcome, women with seroconversion as well as women who were seronegative in the midpregnancy or in the umbilical cord sample were classified as seronegative.

All serological analyses were performed in a certified laboratory at Statens Serum Institut, Denmark. Laboratory personnel were blinded for exposure status and samples were always analyzed in the same batch of commercial kits.

### Statistical analysis

Associations between positive serology (IFA), spontaneous abortion, preterm birth and Small for Gestational Age (SGA) were analyzed by logistic regression. The association between gestational age at birth (which does not follow a normal distribution) and positive IFA serology was tested using a non-parametric (Wilcoxon) test. We examined the association between positive serology (IFA), birth weight and gestational age for children born at term, respectively, by fitting multiple linear regression models.

Maternal age (<25 years, 25–34 years, 35+ years), number of previous pregnancies (0, 1+) and smoking during pregnancy (0, 1–10, 11+ cigarettes per day) were *a priori* selected as potential confounders.

All analyses were carried out using STATA statistical software, version 11.

Women enrolled in the Danish National Birth Cohort gave both verbal and written consent to participate. The women gave permission to include interview information, blood samples, and health information from other registers in the Danish National Birth Cohort. This study was approved by the Danish National Birth Cohort, the Danish Data Protection Board, and the Danish Regional Scientific Ethical Committee.

## Results

Among the 856 women, antibodies against *C. burnetii* (IFA) were detected in 169, while 687 women were IFA negative. The majority (87%) of the IFA positive women had contact to livestock (Table 1).

**Table 1:** Maternal characteristics of pregnant women according to Q fever seropositivity in immunofluorescence antibody test (IFA)

	**IFA positive (n = 169)**	**IFA negative (n = 687)**
**Age**:		
<25 years	10 (5.9%)	94 (13.7%)
25 - <35 years	139 (82.3%)	492 (71.6%)
35 years+	20 (11.3%)	101 (14.7%)
**Prior pregnancies**		
0	57 (33.7%)	250 (36.4%)
1+	112 (66.3%)	437 (64.6%)
**Gestational age at recruitment:**		
<8	21 (12.4%)	111 (16.2%)
Week 8-12	86 (50.9%)	321(46.7%)
Week 12- < 16	38 (22.5%)	186 (27.1%)
Week 16+	24 (14.2%)	69 (10.0%)
**Smoking:**		
Non-smokers:	155 (91.7%)	566 (82.1%)
1- < 10 g/day	4 (2.4%)	64 (9.3%)
+10 g/day	8 (4.7%)	48 (7.0%)
Unknown	2 (1.2%)	9 (1.3%)
**Animal Contact:**		
Occupational or domestic contact to livestock (cattle, goats, sheep)	147 (87.0%)	250 (36.4%)
No contact to livestock	22 (13.1%)	437 (63.6)
**Residence:**		
Living in rural area	121 (71.6%)	303 (44.5%)
Living in non-rural area	45 (26.6%)	373 (54.3%)
Unknown	3 (1.8%)	8 (1.2%)

### IFA positivity

Among the 169 IFA positive women, 159 were positive in IgG phase II; 73 of these were also IgG phase I positive, six were only IgG phase I positive. Seven women were positive in IgM phase II, three in IgM phase I. For six women, there was an overlap in positivity between IgM and IgG phases. Hence, the participants’ serology mainly indicated previous infections.

Maternal age was normally distributed and age at recruitment was similar among IFA positive and IFA negative women (mean: 24.7 years (SD: 7.0) vs. mean: 23 years (SD 9.8)). There was no difference in the number of previous pregnancies between the two groups and the IFA positive and IFA negative women were, on average, recruited at the same gestational age (11 weeks 1 day (SD 3.7) vs. 10 weeks 6 days (SD 3.6)). A higher proportion of IFA negative were smokers. Seropositive samples were mainly from women who had contact to livestock during pregnancy or 3 months prior to becoming pregnant (Table 1).

### Serology and pregnancy outcome

We found no association between positive serology and risk of spontaneous abortion (adjusted OR: 1.5; 95% CI: 0.3-7.6) or preterm birth (adjusted OR: 0.4; 95% CI: 0.1-1.1) (Table 2).

**Table 2:** **Gestational age parameters for Danish pregnant women according to antibodies against *C.burnetii*, immunofluorescence antibody test (IFA)**

	**IFA positive fo*r C. burnetii *antibodies (n = 169)**	**IFA negative for *C. burnetii *antibodies (n = 687)**	**Measures of association**	
			**Crude**	**Adjusted**
**Spontaneous abortion < 22 weeks **(n = 8)	2 (1.2%)	6 (0.9%)	OR: 1.4	1.5 (95% CI: 0.3–7.6)*
**Preterm birth (< week 37) **(n = 41)	3 (1.8%)	38 (5.5%)	OR: 0.3	OR: 0.4 (95% CI: 0.1–1.1)**
**Gestational age (>week 36 + 6) **(n = 806****) Median gestational age (interquartile range)	40 weeks 3 days (39 w, 3d; 41 w,1 d)	40 w, 2 days (39w, 3d; 41w,1d)	Mean difference in days*** 0.91 days	1.2 days (−0.4– +2.7)**

*adjusted for age **adjusted for smoking, age and gravidity *** babies of IFA positive mothers were older **** of which 163 were seropositive and 643 seronegative.

Infants born by seropositive mothers had a 0.9 day older gestational age than infants born by seronegative mothers, but this difference was not significant (p = 0.06, Wilcoxon non-parametric test). The relation between positive IFA serology and gestational age was also tested in a multiple linear regression model which did not change the results significantly (adjusted difference: 1.2 days; 95% CI: -0.4 days - +2.7 days, (Table 2)).

When evaluating the birth weight for all newborns, there was a significant weight difference (168 g; 95% CI: 70-267 g) with the IFA positive babies being heavier; results were similar when restricting analyses to term babies (37 completed weeks or more): (134 g; 95% CI: 47-221 g) (Table 3).

**Table 3:** **Birth weight parameters for Danish pregnant women according to antibodies against *C.burnetii*, immunofluorescence antibody test (IFA)**

	**IFA positive fo*r C. burnetii *antibodies**	**IFA negative for *C. burnetii *antibodies**	**Measures of association**	
			**Crude**	**Adjusted**
**Birth weight all **(n = 842) Median Birth weight (interquartile range)	166	676	Mean difference in gram*: 204 g	168 g (95% CI:70–267 g)**
	3780 g (3480 g; 4085 g)	3600 g (3260 g; 3997 g)		
**Birth weight children born at term** (n = 803***) Median Birth weight (interquartile range)	163	640	Mean difference in gram*: 160 g	134 g (95% CI: 47–221 g)**
	3790 g (3490 g; 4090 g)	3650 g (3309 g; 4000 g)		
**Small for gestational age term pregnancies (week 37+** (n = 82) Median Birth weight (interquartile range)	9	73	OR: 0.5	0.4 (95% CI: 0.8–1.0)**
	2820 g (2275 g; 3010 g)	2850 g (2350 g; 3030 g)		

*IFA positive babies are heavier **adjusted for smoking, age and gravidity.

*** of which 163 were seropositive and 640 seronegative.

We found no association between SGA and seropositivity (IFA) (OR: 0.4; 95% CI: 0.8-1.0) (Table 3).

One IFA negative woman had an induced abortion after pregnancy week 12 due to fetal disease. One preterm birth was a stillbirth in gestational week 23; two women had stillbirths in gestational week 35, all were IFA negative.

To further explore the relationship between contact to livestock, seropositivity and pregnancy outcome, we also examined the pregnancy outcome among IFA positive women with livestock contact compared to IFA negative women with no contact to livestock. We also compared pregnancy outcome among IFA positive versus IFA negative pregnant women within the groups of women with livestock contact. None of the results showed any significant association between seropositivity and adverse pregnancy outcome (not shown).

### Seroconversion and pregnancy outcome

A total of 14 women met the criteria for seroconversion during pregnancy in ELISA. These were confirmatory tested in the IFA; 10 of them seroconverted during pregnancy as defined by the modified Danish cutoff. All had occupational or domestic contact to livestock. All gave live birth at term, however, two newborns were SGA (birth weight: 2110 g and 2236 g, respectively) (Table 4).

**Table 4:** Immunofluorescence antibody test (IFA) titres at beginning and end of pregnancy for the 10 seroconverted pregnancies

	**IFA blood sample beginning of pregnancy**				**IFA blood sample umbilical cord**				**Pregnancy outcome (all live singletons)**	
**Patient**	**IgG phase II**	**IgG phase I**	**IgM phase II**	**IgM phase I**	**IgG phase II**	**IgG phase I**	**IgM phase II**	**IgM phase I**	**Gestatio-nal age, weeks**	**Birth weight**
#1	Neg	Neg	Pos: 1:128	Neg	Pos: 1:2048	Pos: 1:1024	Neg	Neg	38	3190 g
#2	Neg	Neg	Neg	Neg	Pos: 1:256	Pos: 1:128	Neg	Neg	38	4200
#3	Neg	Neg	Neg	Neg	Pos: 1:1024	Pos: 1:512	Neg	Neg	42	2110 g
#4	Neg	Neg	Neg	Neg	Pos: 1:128	Pos: 1:128	Neg	Neg	41	4220 g
#5	Neg	Neg	Neg	Neg	Pos: 1:128	Pos: 1:128	Neg	Neg	41	3430 g
#6	Neg	Neg	Neg	Neg	Pos: 1:128	Pos: 1:128	Neg	Neg	42	3400 g
#7	Neg	Neg	Neg	Neg	Pos: 1:128	Pos: 1:128	Neg	Neg	40	3330 g
#8	Neg	Neg	Neg	Neg	Pos: 1:8192	Pos: 1:256	Neg	Neg	39	2236 g
#9	Neg	Neg	Neg	Neg	Pos: 1:128	Pos: 1:128	Pos: 1:256	Pos: 1:128	40	3520 g
#10	Neg	Neg	Neg	Neg	Pos 1:512	Pos:1:128	Neg	Neg	41	3950 g

None of the seroconverters reported episodes of fever during pregnancy at the interview by the beginning of third trimester.

## Discussion

We hypothesized that being seropositive in pregnancy would be associated with adverse pregnancy outcome, potentially mediated by reactivation of a latent infection [9-11]. We also hypothesized that acute infection during pregnancy would be related to adverse pregnancy outcome. Neither of these hypotheses were confirmed as no increased risk of adverse pregnancy outcome was found in women with verified exposure to *C. burnetii*.

To our knowledge, this is the first population-based seroepidemiologic study evaluating pregnancy outcome in women with serologically verified exposure to *C. burnetii* against a comparable reference group of seronegative women.

When diagnosing Q fever, a variety of serological methods are available; the Panbio ELISA kit has previously been showed to be superior to other methods [18] and suitable for large-scale screening [17,19]. The micro immunofluorescence antibody test (IFA) is regarded as the gold standard [20] because it is capable of determining both phase I and II antibodies simultaneously by the use of two different antigens in a single sample. We have previously demonstrated coherence between ELISA and IFA [21].

Villumsen et al. established a national, very restrictive cutoff in order to obtain a high specificity and a high predictive value of a positive result [21]; this decision was based on the assumption that Q fever was sporadic in Denmark. However, particularly in rural populations of Denmark, Q fever is more widespread than previously considered [7,8] and one may now argue that the cutoff may be too conservative.

Consequently, in the present study, we decided to use a modified version of the Danish cutoff. A more conservative interpretation of the serological values (theoretically leading to a lower positive prevalence and higher predictive value) did not reveal any associations between seropositivity and adverse outcome of pregnancy.

Finally, we also acknowledge that the cutoff applied in our study is high compared with some other studies. However, in a seroepidemiologic study including healthy individuals, our priority was to maintain a high predictive value for a positive result. The application of a lower cutoff would have falsely classified additional women as seropositive and lead to misclassification and thus a higher risk of overlooking a potential association between (true) seropositivity and adverse outcome of pregnancy.

Most of the seropositive women had markers of previous infections, but ten met the criteria for IFA seroconversion. It is worth to note that two out of these women gave birth to infants that were SGA. We cannot draw any conclusions on the risk of adverse pregnancy outcome from 10 cases and the low number of seroconverters is a limitation to this study. Hence, we cannot make an inference with respect to pregnancy outcome in women with acute and, in particular, symptomatic infections.

The risk of reactivation of latent infection leading to adverse pregnancy outcome has been reported [9,10]. However, the IgG positive women in our study had a similar proportion of previous spontaneous abortions as the seronegative women, and overall, reactivation of latent infections leading to adverse pregnancy outcomes was not observed in this population.

Detailed information on previous preterm births was not available, and we chose adjustment for prior pregnancies regardless of pregnancy outcome.

In women with contact to livestock, we had the opportunity to evaluate seroconversion throughout pregnancy; in women with no contact to livestock we only had blood samples from beginning of the pregnancy. This could potentially bias data as the women without animal contact were assumed to be negative throughout pregnancy when, theoretically, they could be infected later in their pregnancy. This is why women with seroconversion as well as women who were seronegative in the midpregnancy or in the umbilical cord sample were classified as seronegative in analyses of pregnancy outcome. Also, stratified analysis on contact to livestock and pregnancy outcome (spontaneous abortion and preterm birth), irrespective of titer status, showed no significant difference between the groups (results not shown).

A high seroprevalence of *C.burnetii* accompanied by few clinical symptoms in farmers and veterinarians has been found in Denmark as well as abroad [7,8,22]. We evaluated pregnancy outcome in seropositive versus seronegative women who had occupational, domestic, or no exposure to livestock (as stated in the methods section). The vast majority of the seropositive women were exposed to animals (Table 1). Due to few unexposed, seropositive women we are unable to study adverse pregnancy outcome in this group of women or clarify whether the dynamics of infection differ in unexposed women compared to women heavily exposed to *C.burnetii*.

The evidence of the impact of Q fever on pregnancy outcome mainly originates from French case studies of referred infected pregnant patients and pregnancies with Q fever diagnosed retrospectively after an adverse pregnancy outcome [7,8]. The authors conclude that there is a link between placentitis and obstetric complications. However, in a recent study by Angelakis et al., [23] a study of 30 pregnant women with acute infection in pregnancy, no placentitis or isolation of *C.burnetii* is found in 14 available biopsies. 17 of the women were asymptomatic, but only two of these had an uncomplicated pregnancy illustrating the difficulty in segregating harmless seroconversion from infection threatening maternal and foetal health. In that study, genotyping showed that QpDV plasmid was present in 4 of 7 *C. burnetii* strains isolated from infected women with miscarriage. Apart from differences in study design, numbers of pregnancies included, selection bias and cutoffs, the disagreements between the French, the Dutch and our studies could be related to strain specificity. Risk assessment and management of Q fever in pregnancy may therefore benefit from further clarification of the role of strain differences and virulence factors.

The present study is subject to some limitations.

Due to the design of the study, it was not possible to include early miscarriage as an outcome. Only few participants were included prior to 8 weeks of gestational age (Table 1). It is possible that the study population is biased towards a “healthy pregnant population”. An increased risk in early pregnancy may in our study be reflected by a “protective” effect in later pregnancy.

Also, maternal IgM cannot be detected in umbilical cord blood, meaning that theoretically we could miss a narrow window of acute infections in very late pregnancy with positive IgM but before IgG phase II elevation; the potential effect on pregnancy outcome from this is, however, speculative.

The French recommendation regarding treatment with cotrimoxazole throughout pregnancy in seropositive women [9,10,23] is widely practiced, but has recently been questioned [24]. However, the number of acute infections in our study is too small to impact these recommendations.

Overall, our findings are in line with two new studies from The Netherlands, a country that recently saw the world’s largest Q fever outbreak [25]. One study included serum samples from early pregnancy of 1174 pregnant women living in the high-risk area and found no association between positive Q fever serology and adverse pregnancy outcome [13]. The other study was a randomized controlled trial with 1229 women split into a screening group and a control group; no difference in pregnancy outcome was found between the two groups [12].

## Conclusion

Seropositivity was not associated with adverse pregnancy outcomes as this study did not find a higher risk of spontaneous abortion, preterm birth, or low birth weight among pregnancies positive for *C. burnetii* compared to seronegative Danish pregnant women.

## Abbreviations

SGA: Small for Gestational Age; ELISA: Enzyme-Linked Immunosorbent Assay; IFA: Immunofluorescence Antibody test; DNBC: the Danish National Birth Cohort.

## Competing interests

The authors declare that they have no competing interests.

## Authors’ contributions

SYN initiated the study, coordinated all blood sample analysis, performed the statistical analysis and drafted the manuscript. AMNA participated in the design of the study and supervised the data collection and management. KRM participated in the design of the study and helped drafting the manuscript. BJK supervised all the ELISA and IFA analysis. KAK helped conceive the study. NHH participated in the design of the study. TBH participated in the design of the study and supervised the statistical analysis. All authors read and approved the final manuscript.

## Pre-publication history

The pre-publication history for this paper can be accessed here:

http://www.biomedcentral.com/1471-2334/13/87/prepub
